# Mutations in the nuclear bile acid receptor FXR cause progressive familial intrahepatic cholestasis

**DOI:** 10.1038/ncomms10713

**Published:** 2016-02-18

**Authors:** Natalia Gomez-Ospina, Carol J. Potter, Rui Xiao, Kandamurugu Manickam, Mi-Sun Kim, Kang Ho Kim, Benjamin L. Shneider, Jennifer L. Picarsic, Theodora A. Jacobson, Jing Zhang, Weimin He, Pengfei Liu, A. S. Knisely, Milton J. Finegold, Donna M. Muzny, Eric Boerwinkle, James R. Lupski, Sharon E. Plon, Richard A. Gibbs, Christine M. Eng, Yaping Yang, Gabriel C. Washington, Matthew H. Porteus, William E. Berquist, Neeraja Kambham, Ravinder J. Singh, Fan Xia, Gregory M. Enns, David D. Moore

**Affiliations:** 1 Lucile Packard Children's Hospital, Divisions of Medical Genetics, Stanford University Medical Center, Stanford, California 94305, USA; 2 Section of Human and Molecular Genetics, Nationwide Children's Hospital, Columbus, Ohio 43205, USA; 3 Department of Molecular and Cellular Biology, Baylor College of Medicine, Houston, Texas 77030, USA; 4 Department of Molecular and Human Genetics, Baylor College of Medicine, Houston, Texas 77030, USA; 5 Department of Pediatrics, Baylor College of Medicine, Houston, Texas 77030, USA; 6 Department of Pathology, University of Pittsburgh School of Medicine, Pittsburgh, Pennsylvania 15261, USA; 7 Institute of Liver Studies, King's College Hospital, London SE5 9R5, UK; 8 Department of Pathology and Immunology, Baylor College of Medicine, Houston, Texas 77030, USA; 9 Human Genome Sequencing Center, Baylor College of Medicine, Houston, Texas 77030, USA; 10 Pediatric Stem Cell Transplantation, Stanford University Medical Center, Stanford, California 94305, USA; 11 Pediatric Gastroenterology, Stanford University Medical Center, Stanford, California 94305, USA; 12 Anatomic and Clinical Pathology, Stanford University Medical Center, Stanford, California 94305, USA; 13 Immunochemistry Core Laboratory, College of Medicine, Mayo Clinic, Rochester, Minnesota 55902, USA

## Abstract

Neonatal cholestasis is a potentially life-threatening condition requiring prompt diagnosis. Mutations in several different genes can cause progressive familial intrahepatic cholestasis, but known genes cannot account for all familial cases. Here we report four individuals from two unrelated families with neonatal cholestasis and mutations in *NR1H4*, which encodes the farnesoid X receptor (FXR), a bile acid-activated nuclear hormone receptor that regulates bile acid metabolism. Clinical features of severe, persistent *NR1H4*-related cholestasis include neonatal onset with rapid progression to end-stage liver disease, vitamin K-independent coagulopathy, low-to-normal serum gamma-glutamyl transferase activity, elevated serum alpha-fetoprotein and undetectable liver bile salt export pump (*ABCB11*) expression. Our findings demonstrate a pivotal function for FXR in bile acid homeostasis and liver protection.

## Abstract (web summary)

Neonatal cholestasis is a result of elevated bile acid levels, and is associated with mutations in genes regulating bile acid homeostasis. Here the authors identify mutations in the bile acid sensing farnesoid X receptor in four individuals with neonatal cholestasis from two unrelated families.

## Graphical abstract

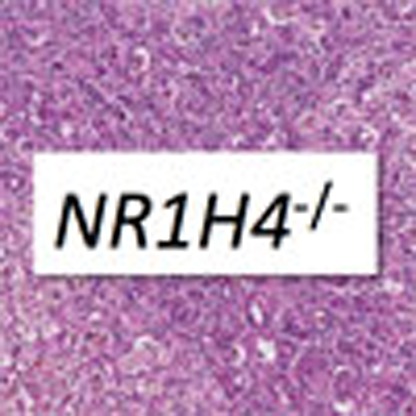

Bile acids are detergents that are produced in the liver and released into the gut to facilitate and promote absorption of lipid nutrients. They are reabsorbed in the gut along with the nutrients and returned to the liver via the portal vein. Excessive bile acid levels are toxic, and both their hepatic production and enterohepatic circulation are stringently regulated. Bile acid levels are sensed by a nuclear bile acid receptor termed the farnesoid X receptor (FXR), encoded by the *NR1H4* gene. Elevated bile acid levels activate FXR to induce a program that represses hepatocyte bile acid biosynthesis and uptake while increasing export^1^,^2^.

This suppression of bile acid production makes FXR a therapeutic target for cholestatic liver disease, and clinical trials of FXR agonists in primary biliary cirrhosis are promising^3^,^4^,^5^. FXR activation suppresses autophagy^6^ and affects additional metabolic targets outside of bile acid homeostasis, and FXR ligands have beneficial effects in mouse models of metabolic disease^7^,^8^. In accord with this, FXR has been identified as a potential mediator of the rapidly beneficial metabolic effects of bariatric surgery in mice^9^, and initial clinical results indicate that FXR agonists may have beneficial effects in non-alcoholic fatty liver disease and the metabolic syndrome^10^,^11^. Thus, FXR is both a key modulator of multiple metabolic and hepatocyte-protective pathways and an emerging therapeutic target for both cholestatic and metabolic diseases.

Mutations in several genes encoding proteins involved in bile acid homeostasis cause neonatal cholestasis. Progressive familial intrahepatic cholestasis (PFIC) types 1, 2 and 3 are a group of cholestatic conditions caused by mutations in *ATP8B1, ABCB11* and *ABCB4* respectively, and defects in *TJP2*, encoding tight junction protein 2, can also cause severe cholestatic liver disease^12^. Multiple heterozygous variants in known PFIC genes may contribute to disease in some cases^13^, but up to one third of cases remain idiopathic^14^. Patients with PFIC1 or PFIC2 and patients with *TJP2* mutations characteristically present with low-to-normal serum gamma-glutamyl transferase (GGT) activity^12^,^15^. Both *ABCB11*, which encodes the bile salt export pump (BSEP) and is deficient in PFIC2, and *ABCB4,* which encodes multidrug resistance protein 3, MDR3, and is deficient in PFIC3, are direct targets of FXR. Variants of *NR1H4*, located at 12q23.1, have been associated with increased risk for intrahepatic cholestasis of pregnancy^16^. A recent search for functional *NR1H4* variation in cholestatic patients found none^17^, but a heterozygous *NR1H4* variant has been reported in one patient with infantile cholestasis^18^. Here we describe four patients from two families with homozygous loss of *NR1H4* function and severe neonatal cholestasis.

## Results

Whole-exome sequencing and single-nucleotide polymorphism (SNP) arrays of two unrelated probands with severe cholestasis revealed homozygous loss of function mutations in *NR1H4*, located at 12q23.1. Further analysis confirmed the same *NR1H4* genotype for an additional affected individual in each family (Table 1). All patients presented with liver dysfunction at an early age (Table 1). Three presented with neonatal cholestasis; patient 4 presented with ascites, pleural effusions and intraventricular haemorrhage at birth. At the time of initial evaluation all patients had conjugated hyperbilirubinemia, elevated aminotransferases, low-to-normal GGT and elevated prothrombin time and international normalized ratio (Table 1 and Supplementary Table 1). Factors V and VII levels in patients 1 and 3 and were markedly reduced. Alpha-fetoprotein, measured in three patients, was strikingly increased early in the course of the disease and trended down with time. Serum bile acids were elevated in patient 1. Patients 1–3 developed liver failure in the first 2 years of life with worsening coagulopathy, hypoglycemia and hyperammonemia. Both patients in family 1 underwent deceased donor liver transplants, while patient 3 died awaiting transplantation. Patient 4 died at 5 weeks from complications from an aortic thrombus.

**Figure 1:**
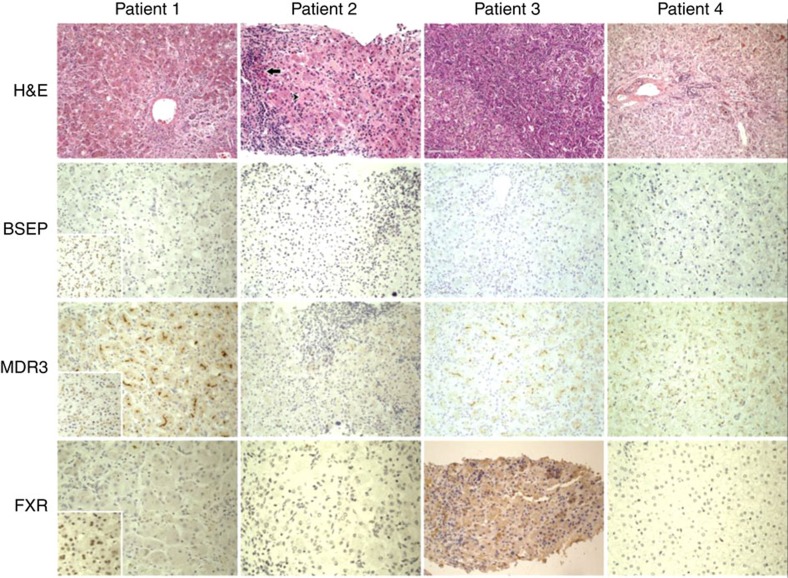
Histological and immunohistochemical findings in *1HR1H4* mutant livers. Expression of BSEP, MDR3 and FXR in liver (diaminobenzidine chromogen and hematoxylin counterstain) in patients with *NR1H4*-related cholestasis; normal controls in insets. Hematoxylin/eosin views for orientation. Original magnification, all images, × 200. BSEP and FXR are absent in all four patients when compared with positive control (inset). MDR3 is present in all four patients although reduced in patients 2 and 4. H&E, hematoxylin and eosin.

Histological examination of the liver in all four patients showed ductular reaction, diffuse giant cell transformation and ballooning of hepatocytes and intralobular cholestasis (Fig. 1). The degree of inflammatory cell infiltration and fibrosis varied with the progression of the disease, but the explanted liver of patient 1 showed micronodular cirrhosis and fibrosis was evident at later stages for all patients. Autopsy of patient 3 revealed siderosis in the liver, pancreas, heart and thyroid gland (Supplementary Fig. 1) suggesting gestational alloimmune liver disease, but immunohistochemical studies did not support this diagnosis^19^. Liver immunohistochemistry showed no detectable expression of BSEP in bile canaliculi in all patients, but expression of the phospholipid transporter MDR3 was preserved (Fig. 1). Additional immunohistochemical studies in patient 1 showed normal expression of alanyl aminopeptidase, GGT and MRP2, as well as increased perisinusoidal and perihepatocytic reticulin (Supplementary Fig. 2). Although these results suggested *ABCB11* deficiency, sequencing and deletion–duplication analysis of *ABCB11* in patient 1 was negative.

**Figure 2:**
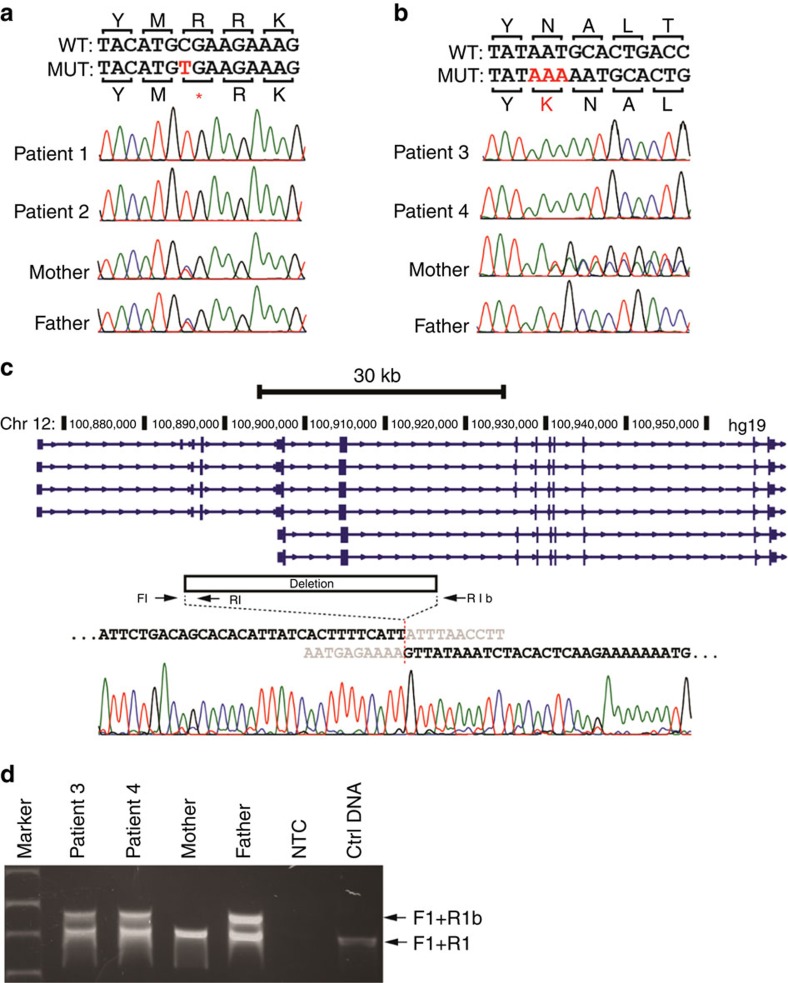
*NR1H4* mutations. (**a**) Sanger sequencing of the homozygous variant c.526C>T (p.R176*) in patients 1 and 2 of family 1. Both parents are heterozygous carriers. (**b**) Sequence of the homozygous variant c.419_420insAAA (p.Tyr139_Asn140insLys) in patients 3 and 4 of family 2. The mother carries a heterozygous change. (**c**) Breakpoint junction mapping in family 2. PCR and Sanger sequencing confirmed a 31.7 kb deletion that spans the first two coding exons of all *NR1H4* isoforms. The deletion region is marked with a filled box. (**d**) All family 2 members except the mother carry this deletion. PCR was performed using forward and reverse primers indicated with arrow heads in **d**. Primer pair F1/R1b fails to amplify at the wild-type locus because the distance between them exceeds the limit of PCR reaction. Primer pair F1/R1 serves as control. NTC, no template control.

Whole-exome sequencing of patient 2 (family 1) identified an apparently homozygous c.526C>T (p.R176*) mutation in *NR1H4* that prematurely terminates the protein at amino acid 176 (nucleotide: NM_005123 and protein: NP_005114) in the DNA-binding domain (DBD). The same homozygous mutation was found in patient 1; both asymptomatic parents are carriers (Fig. 2a and Table 2). Consanguinity in this family was confirmed by cSNP array detection of ∼89 Mb of total homozygous regions larger than 5 Mb each in patient 1. The predicted residual protein fragment lacks both DNA binding and hormone receptor domains, and immunohistochemistry showed no FXR expression (Fig. 1). This variant has been reported previously in one patient with infantile cholestasis as a heterozygous change^18^ but the heterozygous family 1 parents had normal liver biochemistry. The mother did not have symptoms of cholestasis in any of her three pregnancies.

Whole-exome sequencing of patient 4 (family 2) identified an apparently homozygous in-frame insertion variant c.419_420insAAA (p.Tyr139_Asn140insLys) in *NR1H4*, which was also demonstrated in patient 3 (Fig. 2b). Despite the expected expression of the in-frame insertion mutant product, FXR protein was undetectable in both patients (Fig. 1). The mother carried the in-frame insertion in *NR1H4* and the father was negative. A SNP array suggested copy number loss (Supplementary Fig. 3), and breakpoint junction PCR showed that both patients carry a paternally inherited 31.7 kb deletion within the *NR1H4* gene (Fig. 2c,d). This deletion spans the first two coding exons of all transcripts of *NR1H4* and is predicted to produce a complete loss of FXR expression. Patient 4 was also heterozygous for a c.197G>A (p.Trp66*) predicted pathogenic variant in *SLC10A2*, encoding the ileal apical sodium-dependent bile acid transporter (ASBT). This variant was absent in patient 3 (Table 2). Patient 4 was also found to be homozygous for the common c.1331T>C (p.V444A) variant in *ABCB11*. Patient 3 was heterozygous for this allele, which was not present in family 1 (Table 2).

**Figure 3:**
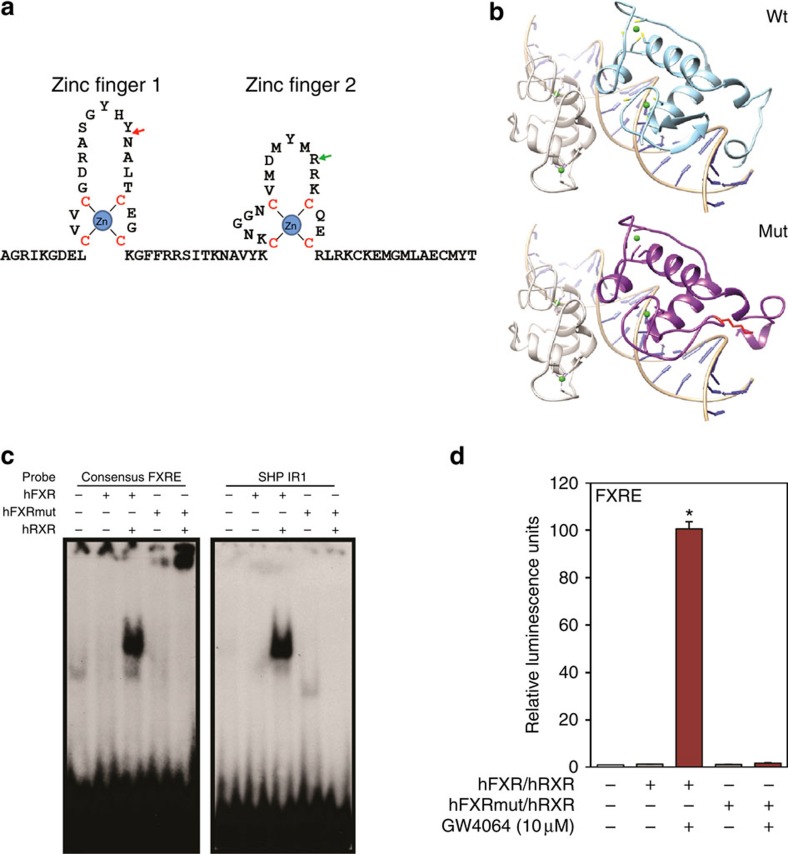
FXR Lys insertion mutation abrogates consensus IR-1 FXR binding and transactivation. (**a**) The highly conserved zinc-finger domain of the human FXR protein. The residue of nonsense mutation found in family 1 is indicated with a green arrow. The location of the in-frame Lys 140 insertion found in family 2 is indicated with a red arrow. ‘Zn' stands for zinc ions. Cysteine residues that directly interact with zinc ions are in red. (**b**) Comparison of predicted structures of DBD. Predicted structures of wild-type FXR and FXR mutant with in-frame insertion based on the structure of EcR/RXR DBD in complex with IR-1 (PBD 1R0N). RXR is rendered in grey, EcR in orange, wild-type hFXR in cyan, hFXR mutant in purple and zinc ions are in green. The inserted lysine residue is in red with side chain shown. (**c**) Electrophoretic mobility-shift assay performed with a consensus FXRE or the FXR-binding region in mouse *SHP* promoter and wild-type or Lys insertion mutant FXR and RXR as indicated. (**d**) Relative luciferase expression directed by a reporter containing three copies of a consensus in response to wild-type and mutant FXR proteins in the presence or absence of the synthetic FXR agonist GW4064. Repeated three times (*P, 0.01 by *t*-test, error bars s.d.). FXRE, FXR-binding element.

The c.419_420insAAA (p.Tyr139_Asn140insLys) insertion is located in the first zinc-binding module of the FXR DBD close to the DNA-recognition helix (Fig. 3a,b). This region of the DBD is highly conserved in vertebrate nuclear receptors (Supplementary Fig. 4), suggesting that this insertion disrupts DNA binding. Although expression of the mutant protein was not detected immunohistochemically, it might be expressed at low levels. Therefore, both wild-type and insertion mutant FXR were produced by *in vitro* translation, along with the retinoid X receptor (RXRα) heterodimer partner, and FXR/RXR DNA binding was assessed using an electrophoretic mobility-shift assay. The mutant FXR showed no detectable binding to a consensus FXR/RXR-binding site containing an inverted repeat of the receptor-binding hexamer separated by one base pair (IR-1) or the IR-1 element from the *SHP* promoter (Fig. 3c). The insertion also abolished transcriptional activity in response to the synthetic agonist GW4064 on a synthetic promoter containing three copies of a consensus IR-1 (Fig. 3d). In addition to this IR-1 transactivation, we have recently reported that FXR can transcriptionally repress expression of autophagic genes via a distinct DR-1 direct repeat element^6^. The mutant protein was also defective in this function (Supplementary Fig. 5).

In addition to the liver, FXR is highly expressed in the small intestine where it regulates FGF19, a hormonal growth factor that acts in the liver to decrease bile acid synthesis by repressing expression of the biosynthetic enzyme CYP7A1 (ref. 20). Consistent with absent intestinal FXR, the surviving family 1 patients have low serum FGF19 (31.6±6 pg ml^−1^; pediatric normal 220±35 (ref. 21)) accompanied by high CYP7A1 activity, as evidenced by high serum levels of the bile acid precursor C4 (211.8±124; pediatric normal 22.8±15.8 ng ml^−1^ (ref. 22)), and low cholesterol (63±8 mg dl^−1^) (Supplementary Fig. 6d–f). These patients also have persistent serum bilirubin and liver enzyme abnormalities (Supplementary Fig. 6a–c) and histological evidence of progressive steatosis. It is possible that their mild post-transplant hepatopathy is a consequence of the disruption of normal enterohepatic bile acid homeostasis. Post-transplant progressive hepatic steatosis has also been reported for PFIC1 (refs 23, 24), perhaps due to altered FXR signaling^25^.

All patients presented with severe vitamin K-independent coagulopathy early in the course of their disease. This is consistent with a recent genome-wide ChIP-Seq DNA binding study that identified the complement and coagulation cascades as direct regulatory targets of FXR in human primary hepatocytes^26^, and also with earlier reports that human FXR induces expression of fibrinogen^27^ and kininogen^28^. We confirmed that FXR agonists induce mRNA expression of all three fibrinogen genes *FGA*, *FGB* and *FGG*, and also increase expression of the genes encoding tissue factor/factor III/thromboplastin (*F3*), factor XII/Hageman factor (*F12*), and factor XIII B (*F13B*) in the human liver cell line Huh7 (Supplementary Fig. 7a). These responses were decreased by FXR knockdown (Supplementary Fig. 7b).

## Discussion

We conclude that homozygous loss of FXR function due to *NR1H4* mutations causes a low GGT form of severe PFIC. The clinical features of *NR1H4*-related cholestasis include neonatal onset with rapid progression to end-stage liver disease, early onset vitamin K-independent coagulopathy, low-to-normal serum GGT, elevated serum alpha-fetoprotein and undetectable liver BSEP expression. The lack of elevation of GGT is consistent with the loss of BSEP expression. BSEP, encoded by *ABCB11*, was the first identified FXR target gene. The BSEP promoter is particularly responsive to FXR activation^29^, suggesting that the absence of BSEP expression is a direct consequence of the loss of FXR function. Expression of MDR3, which is less responsive to FXR^30^, was retained.

The basis for the more severe symptoms of both patients in family 2, and the hydropic presentation in patient 4, is not clear. Homozygous loss of ASBT/*SLC10A2* function causes bile acid malabsorption as early as the second day of life^31^ and ASBT haploinsufficiency could further disrupt bile acid homeostasis in the context of an increased prenatal bile acid pool. Thus, heterozygosity for a pathogenic variant in the *SLC10A2* gene could contribute to the severe pathology of patient 4. This patient was also homozygous for a common *ABCB11* c.1331T>C (p.V444A) variant, which has been associated with increased risk for several forms of intrahepatic cholestasis^32^. However, the lack of detectable BSEP protein expression in all four patients argues against a functional impact of this polymorphism in patients 3 or 4. The absence of detectable FXR expression also argues against a potential dominant negative impact of the p.Tyr139_Asn140insLys insertion mutant protein.

Since both family 1 parents are heterozygous for the p.R176* truncation but do not show liver dysfunction, our results are not consistent with a prior report of severe infantile cholestasis in an individual heterozygous for the same mutation^18^. Even if the short mutant peptide were expressed, it lacks both the DBD and ligand-binding domain and there is no obvious mechanism for it to exert a dominant negative effect. This simple loss of function would therefore be expected to be recessive to the wild-type allele, as observed for both of the family 1 parents, both of the heterozygous family 2 parents, and also in mice lacking a single *NR1H4* allele. One possibility is that the proband in the prior report harboured an additional, undetected mutation affecting the other *NR1H4* allele. However, this patient had elevated serum GGT levels^18^, which is not consistent with the four individuals presented here with confirmed biallelic loss of *NR1H4*. Thus, the earlier patient may have had a deleterious mutation in another gene associated with cholestasis, as observed in patient 4, or more generally some other problem that compromised liver function. Overall, it is apparent that heterozygous loss of FXR function is not sufficient to cause infantile cholestasis.

The coagulopathy observed in all four patients could be interpreted as a simple consequence of their liver disease or could be secondary to a problem with absorption of lipid-soluble nutrients, particularly vitamin K, which is essential for clotting. However, four distinct but complementary lines of evidence argue that it is a direct consequence of the loss of FXR function. The first is that coagulopathy was observed very early in the course of their disease, well before end-stage liver failure. This early onset coagulopathy was not responsive to vitamin K treatment. The second is that several prior studies have suggested such a function for FXR. Initial reports indicating that human FXR induces expression of fibrinogen^27^ and kininogen^28^ were strongly reinforced by a more recent ChIP-Seq analysis of the FXR cistrome in human primary hepatocytes, which identified multiple components of the complement and coagulation cascades as specifically enriched FXR targets^26^. We confirmed FXR-dependent induction of all three fibrinogen genes and at least three additional coagulation factors in a human liver cell line (Supplementary Fig. 7). The third is an increase in coagulation rate in patients treated with the FXR agonist obeticholic acid, as indicated by a modest but significant decrease in the international normalized ratio^11^. In accord with this, the fourth is a recent report of increased coagulability in patients with a range of cholestatic disorders^33^. This was considered paradoxical from the perspective of liver failure, but in the current context is a directly predicted consequence of FXR activation by elevated bile acids. Consistent with this characterization of a broader range of patients, vitamin K-independent coagulopathy is not a common early finding for the PFIC group of disorders^34^. Thus, our patient data, the direct role of FXR in regulating coagulation, and clinical studies in obeticholic acid treated patients and in other forms of cholestasis all suggest early onset, vitamin K-independent coagulopathy as a distinguishing diagnostic feature of *NR1H4*-reated cholestasis.

The surviving patients in family 1 are genetically mosaic, with wild-type FXR expressed only in the liver. Since they do not show obvious pathology in other organs known to express FXR, including intestine and kidney, such extrahepatic FXR function is not essential. However, it is likely that the absence of FXR in the intestine accounts for their very low levels of circulating FGF19. In turn, the loss of this negative feedback regulator likely accounts for their increased bile acid synthesis, moderately elevated liver enzymes and hepatic steatosis.

In contrast to the severe disease of the human patients, loss of FXR function in mice results in only a modest elevation of bile acid levels that does not cause liver failure^35^,^36^. One potential explanation for this is that mice have an additional FXR isoform, FXRβ, encoded by *NrIh5* (ref. 37). *NR1H5* is a pseudogene in humans, with multiple in frame stop codons and also frame shifts in the predicted transcript. Although very little is known about the function of FXRβ, it is likely that it overlaps with that of FXRα/*NrIh4*. Thus, it is possible that FXRβ expression partially compensates for the loss of FXRα/*NrIh4* in mice.

Overall, the development of early cholestasis and rapidly progressive hepatic dysfunction in humans with mutations in the *NR1H4* gene reinforces the role of FXR as a master regulator of bile acid homeostasis, and confirms a key role in hepatoprotection by preventing bile acid-induced liver toxicity. We predict that some of the patients that show PFIC characteristics with normal GGT and without identified mutations in known PFIC genes^12^,^34^ will carry loss-of-function variants in *NR1H4*, and that these patients may be clinically distinguished by their early onset, vitamin K-independent coagulopathy and rapid progression.

## Methods

### Clinical whole-exome sequencing

Subjects were enrolled in research studies approved by the institutional review boards of Baylor College of Medicine or Stanford University School of Medicine, and informed consent was obtained. Clinical whole-exome sequencing was performed as described previously^38^. Briefly, genomic DNA samples were fragmented by sonication, ligated to multiplexing paired-end adapters, amplified by PCR and hybridized to a solution-based exome capture library that was designed in-house. Enriched DNA products were paired-end sequenced (100 bp) on an Illumina HiSeq 2000 platform, with a mean coverage of > × 130 and at least × 20 coverage for >95% of the target regions. The data were analysed and annotated by a pipeline developed in-house. All samples underwent cSNP array analysis performed with human exome BeadChip (Illumina, San Diego, CA) according to manufacturer's instructions.

### Breakpoint junction PCR

To map the junction of the deletion, serial pairs of primers were designed with about 15 kb intervals that flanked the estimated minimal deletion region as defined by cSNP array result. PCR was performed with Qiagen LongRange PCR kit (Qiagen, Valencia, CA). This strategy yielded amplicons no more than 20 kb in length, ensuring PCR amplification failure without inter-pair deletion. With successful amplification, addition primer sets were designed within that region to generate PCR products less than 1,000 bp in length for Sanger sequencing confirmation.

### Protein sequence alignment and structure prediction

Protein sequences were retrieved from UniProt (www.uniprot.org). Sequence alignment was performed with Clustal X (ref. 39). Predictive structures of wild type FXR and FXR mutant with in-frame insertion were generated with RaptorX (ref. 40) and compared with the structure of EcR/RXR DBD in complex with IR-1 (PBD 1R0N) (ref. 41).

### Electrophoretic mobility-shift assay

Electrophoretic mobility-shift assay was performed as described^5^ with modifications as below. Double-stranded oligonucleotides (consensus FXRE/IR1 and SHP/IR1, 5′-GATGGGCCAAGGTCAATGACCTCGGGG-3′ and 5′-CCCTGGTACAGCCTGGGTTAATGACCCTGTTTATGCACTTG-3′, respectively) were end-labelled with [γ-32P] ATP (3,000 Ci per mmol) using T4 polynucleotide kinase for probe labelling. Human FXR and RXRα proteins (Fig. 2c) were prepared with a TNT T7 Quick coupled system (Promega, Madison, WI). *In vitro* translated proteins were incubated with 0.05 ng of probe in the binding reaction buffer containing 10 mM Tris (pH 8.0), 60 mM KCl, 0.1% NP-40, 6% glycerol, 1 mM dithiothreitol and1 μg of poly(dI-dC) on ice for 20 min. DNA-protein complexes were resolved on standard non-denaturing 4% polyacrylamide gels. The gels were dried and exposed to X-ray film at −80 °C for 5–24 h.

### FXR transactivation assay

Hela cells and AML12 cells were maintained in DMEM with 10% fetal bovine serum (FBS) at 37 °C, 5% CO2. For Hela cell transfection, cells were seeded to 12-well plates and transfected with 1 μg reporter vector (pGL3-FXREx3) and 50 ng per 100 ng of pCMX-hFXR, pCMX-hFXR mutant, pCMX-hRXRα, respectively, using iM Fectin (GenDEPOT, Barker, TX) according to manufacturer's instructions. For internal control, 10 ng of beta-galactosidase plasmid was cotransfected. Three hours after transfection, cells were treated with vehicle (dimethyl sulfoxide) or 1 μM synthetic FXR agonist GW4064 (Sigma-Aldrich, St Louis, MO) and incubated for additional 24 h. Finally cells lysates were collected and luciferase activities were measured as described previously^42^. For AML12 transfection, cells were seeded to 24-well plates and transfected with 200 ng of reporter vector (pGL3-DR1x3 and pGL3-LC3ax3), 100 ng of p(CMX)-mPPARα, or pCMX- hFXR, and 50 ng of CMX-β-galactosidase. After 4 h transfection, cells were treated with or vehicle (0.1% DMSO) using Lipofectamine 2000 (Invitrogen), 10 μM Wy-14,643 or 1 μM GW4064 in media containing charcoal-stripped serum for 20 h before performing luciferase and β-galactosidase assay.

### siRNA transfection

Huh7 cells were maintained in DMEM supplemented with 10% FBS (Life technologies). For ligand treatment, media were replaced with phenol red-free DMEM supplemented 5% charcoal-stripped FBS (Life technologies) then FXR ligands (CDCA, Sigma, C-9377; GW4064, Tocris bioscience, #2473) were treated for 24 h. The siRNAs targeting human FXR (Dharmacon, L-003414-00-0005) were transfected with Lipofectamine 2000 (Life technologies) according to the manufacturer's protocol. After 24 h, media were replaced with phenol red-free DMEM containing 5% chacoal-stripped FBS then FXR ligands were treated for following 48 h.

### RNA isolation and real-time PCR analysis

Total RNA was isolated using Quick-RNA MiniPrep Kit (Zymo Research, #11-328) and cDNA was prepared using amfiRivert cDNA Synthesis Platinum Master Mix (GenDEPOT, #5600). Gene expression level was determined by real-time PCR using LightCycler 480 Real-Time PCR System (Roche) with PerfeCTa SYBR Green FastMix (Quanta Biosciences). Expression values were normalized to GAPDH mRNA. Primer sets are listed in Table 3.

### FGF19 measurements

Serum FGF19 was measured on frozen fasting serum samples from patients 1 and 2 and healthy young adults by quantitative sandwich enzyme-linked immunosorbent assay, using a commercially available kit (FGF19 Quantikine ELISA kit, Cat No. DF1900; R&D Systems, Minneapolis, MN), and following the manufacturer's instructions. We performed two independent measurements where 100 μl of serum were used and all samples were assayed in triplicate.

### Measurement of serum 7-alpha-hydroxy-4-cholesten-3-one (C4)

C4 was measured in frozen fasting serum samples from patients 1 and 2 and healthy young adults. Measurements were determined using a high-performance liquid chromatography-tandem mass spectrometry as previosuly described^43^.

## Author contibutions

N.G.-O., C.J.P., R.X. F.X., B.L.S., G.M.E. and D.D.M. conceived, designed and directed the study. N.G.-O., C.J.P., B.L.S., K.M., J.L.P., T.A.J., G.C.W., M.P., W.E.B., R.J.S. and N.K. saw patients or collected or analysed patient samples. R.X and F.X. designed, performed and interpreted genetic analyses, with substantial contributions from J.Z., W.H., P.L., D.M.M., E.B., J.R.L., S.E.P., R.A.G., C.M.E. and Y.Y. A.S.K. and M.J.F. carried out histological analysis. N.G-O., R.X. and D.D.M. wrote the manuscript, with substantial contributions from C.J.P, B.L.S, A.S.K. and M.J.F. All authors approved the final manuscript and contributed to its intellectual content.

## Additional information

**Accession codes:** Whole exome sequencing data for the pathogenic *NR1H4* variants have been deposited in the NCBI ClinVar database under accession codes SCV000258398, SCV000258399 and SCV000258400.

**How to cite this article:** Gomez-Ospina, N. *et al.* Mutations in the nuclear bile acid receptor FXR cause progressive familial intrahepatic cholestasis. *Nat. Commun.* 7:10713 doi: 10.1038/ncomms10713 (2016).

## Supplementary Material

Supplementary Information Supplementary Figures 1-7 and Supplementary Tables 1-2

## Figures and Tables

**Table 1:** Summary of clinical and laboratory findings in individuals harbouring mutations in *NR1H4*.

	**Family 1**		**Family 2**	
	**Patient 1**	**Patient 2**	**Patient 3**	**Patient 4**
Sex	Female	Male	Female	Male
Age at onset	2 Weeks	2 Weeks	6 Weeks	Birth
Age at initial evaluation	20 Months	7 Weeks	6 Weeks	Birth
Age at liver transplant	22 Months	4.4 Months	NA	NA
Age at last evaluation	10 Years	15 Months	Died at 8 months	Died at 5 weeks
Symptoms	Jaundice, FTT	Jaundice, FTT	Jaundice	Hydrops, IVH

**Liver biochemistry**	**Initial evaluation**	**Before OLT**	**Initial evaluation**	**Before OLT**	**Initial evaluation**	**Before death**	**Birth to death**
Direct bilirubin (nl <0.4 mg dl^−1^)	11.5	14.1	11.8	16.5	9.2	15.7	0.2–13.5
AST (nl <80 U l^−1^)	231	182	392	316	293	116	83–341
ALT (nl <80 U l^−1^)	138	90	219	132	153	62	51–548
GGT (nl <2 years <100 U l^−1^; >2 years <60 U l^−1^)	53	41	45	44	59	38	(107*)
AFP (nl 8–468 ng ml^−1^)	716	NM	146,000	400,000	NM	139,000	NM
							
*Coagulation parameters*							
PT (nl 10.3–13.4 s)	23.9	26	21.3	28.3	17.4	57.4	†
INR (nl 0.9–1.1)	2.0	2.2	2.0	2.8	1.4	7.4	2.05†
Factor V assay (79–127%)	31	NM	NM	NM	45	NM	NM
Factor VII assay (51–116%)	14	NM	NM	NM	32	NM	NM

AFP, alpha-fetoprotein; ALT, alanine aminotransferase; AST, aspartate aminotransferase; FTT, failure to thrive; GGT, gamma-glutamyl transferase activity; INR, international normalized ratio; IVH, intraventricular haemorrhage; NM, not measured; NA, not applicable; nl, normal; OLT, orthotopic liver transplant; PT, prothrombin time.

Patients 1 and 2 were from consanguineous parents, while patients 3 and 4 were not from consanguineous parents.

^*^5-day-old sample—normal range for the GGT assay for patient 4 was 25–148 U l^−1^.

^†^The values obtained were not representative because of transfusions required for volume/losses and blood pressure support.

**Table 2:** Relevant genetic findings in patients harbouring mutations in *NR1H4*.

	**Family 1**		**Family 2**	
	**Patient 1**	**Patient 2**	**Patient 3**	**Patient 4**
*Genetics*				
* NRIH4* first allele	c.526C>T p.Arg176*	c.526C>T p.Arg176*	c.419_420insAAA (p.Tyr139_Asn140insLys)	c.419_420insAAA (p.Tyr139_Asn140insLys)
* NRIH4* second allele	c.526C>T p.Arg176*	c.526C>T p.Arg176*	31.7 kb deletion	31.7 kb deletion
* ABCB11*	None found	None found	Heterozygous c.1331T>C p.V444A	Homozygous c.1331T>C p.V444A

**Table 3:** Primer sets for real-time PCR analysis.

**Gene**	**Forward**	**Reverse**
*GAPDH*	5'-CCAGCAAGAGCACAAGAGGA-3'	5'-GAGATTCAGTGTGGTGGGGG-3'
*FXR*	5'-GCTTTGCTGAAAGGGTCTGC-3'	5'-CAGAATGCCCAGACGGAAGT-3'
*SHP*	5'-TCAAGTCCATTCCGACCAGC-3'	5'-AAGAAGGCCAGCGATGTCAA-3'
*FGF19*	5'-AGATCAAGGCAGTCGCTCTG-3'	5'-CGGATCTCCTCCTCGAAAGC-3'
*BSEP*	5'-GAGCCTGGTCATCTTGTGCT-3'	5'-TCTCCAGGGCCTGCTTATCT-3'
*FGA*	5'-CTGCCTGGTCCTAAGTGTGG-3'	5'-GCAGAAGGGCCAGTCTGAAT-3'
*FGB*	5'-ACCATGGAAAAGCCACCACT-3'	5'-CGTTTCTATGGGCAACAGGC-3'
*FGG*	5'-CACACAGTCTGCCATCCCATA-3'	5'-ACTTGTCAGCTTCAGGTCCC-3'
*F3*	5'-ACTCCCCAGAGTTCACACCT-3'	5'-TCCCGGAGGCTTAGGAAAGT-3'
*F12*	5'-AGCTGAAGAGCACACAGTCG-3'	5'-CGGCCCTTGTGGGTACATTT-3'
*F13b*	5'-GAATGCAATGTGACAGAGGGC-3'	5'-TGACTCCTCTTTCTGCCATTCA-3'
