# Supplementary material for: Mutations in the nuclear bile acid receptor FXR cause progressive familial intrahepatic cholestasis
Source: Nat Commun. 2016 Feb 18;7:10713. doi: 10.1038/ncomms10713 (PMC4759630; doi:10.1038/ncomms10713)
Supplement: Supplementary Information — Supplementary Figures 1-7 and Supplementary Tables 1-2 [file ncomms10713-s1.pdf]

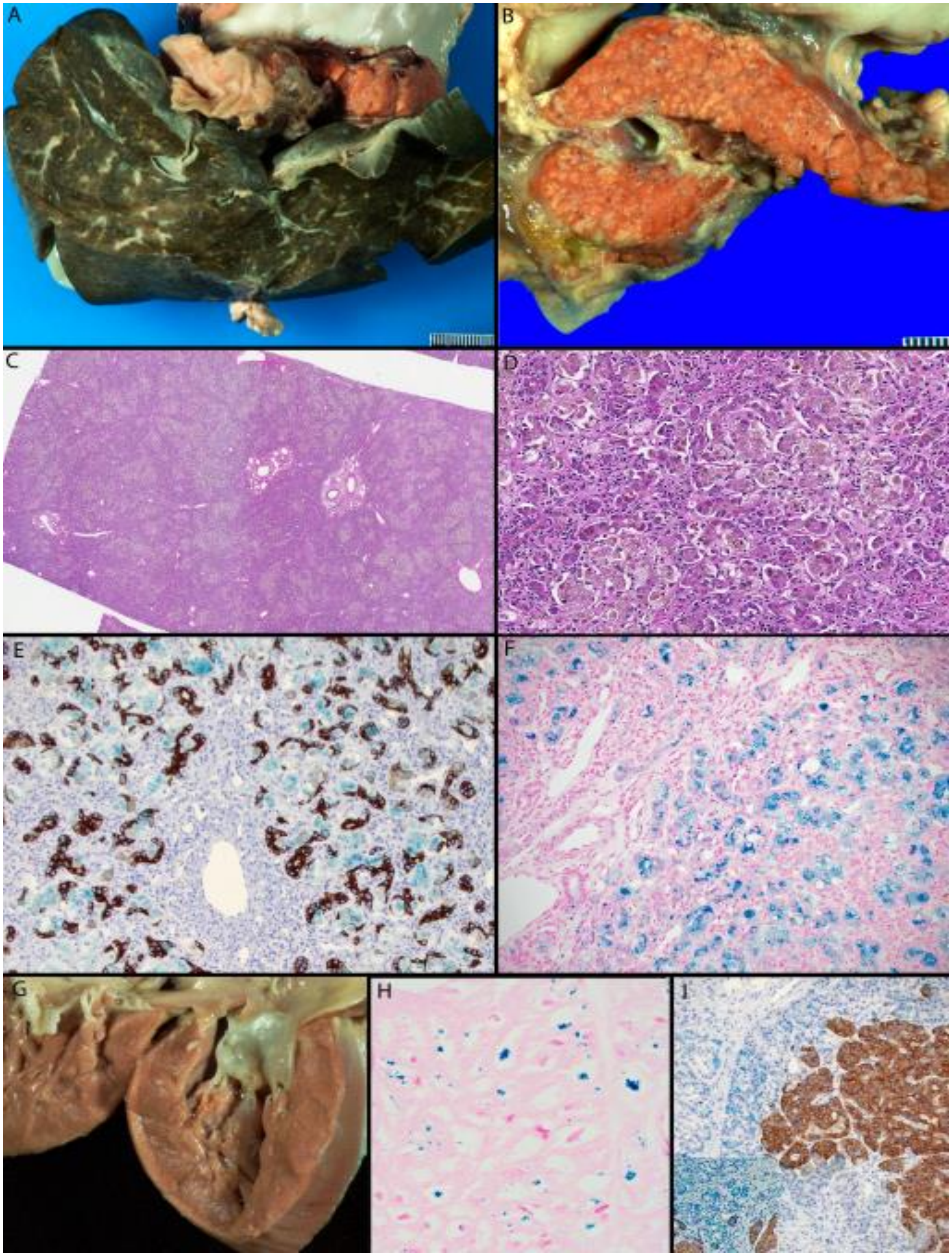

Supplementary Figure 1. Postmortem findings patient 3. **A.** Diffusely cholestatic liver at autopsy. **B.** Enlarged pancreas with yellow-orange lobular parenchyma. **C-D.**

Liver with giant cell transformation and ballooning hepatopathy with intrahepatic cholestasis and ductular proliferation (**C.** H&E, low magnification, **D.** H&E 400x magnification). **E.** Cytokeratin 7 highlights extensive ductular proliferation and transformation of the hepatocytes with intrahepatic siderosis highlighted by the dual counter iron stain (Immunohistochemistry 10x). **F**. Iron stain highlights extensive hemosiderosis of the liver (Prussian blue stain 20x). **G.** Left ventricular concentric hypertrophy (0.9 cm wall thickeness). **H.** Iron stain shows mild siderosis in swollen cardiomyocytes (Prussian blue 100x). **I.** Synaptophysin highlights pancreatic endocrine hyperplasia and mild siderosis by the dual counter iron stain (inlet) of the exocrine acini (Immunohistochemistry 20x).

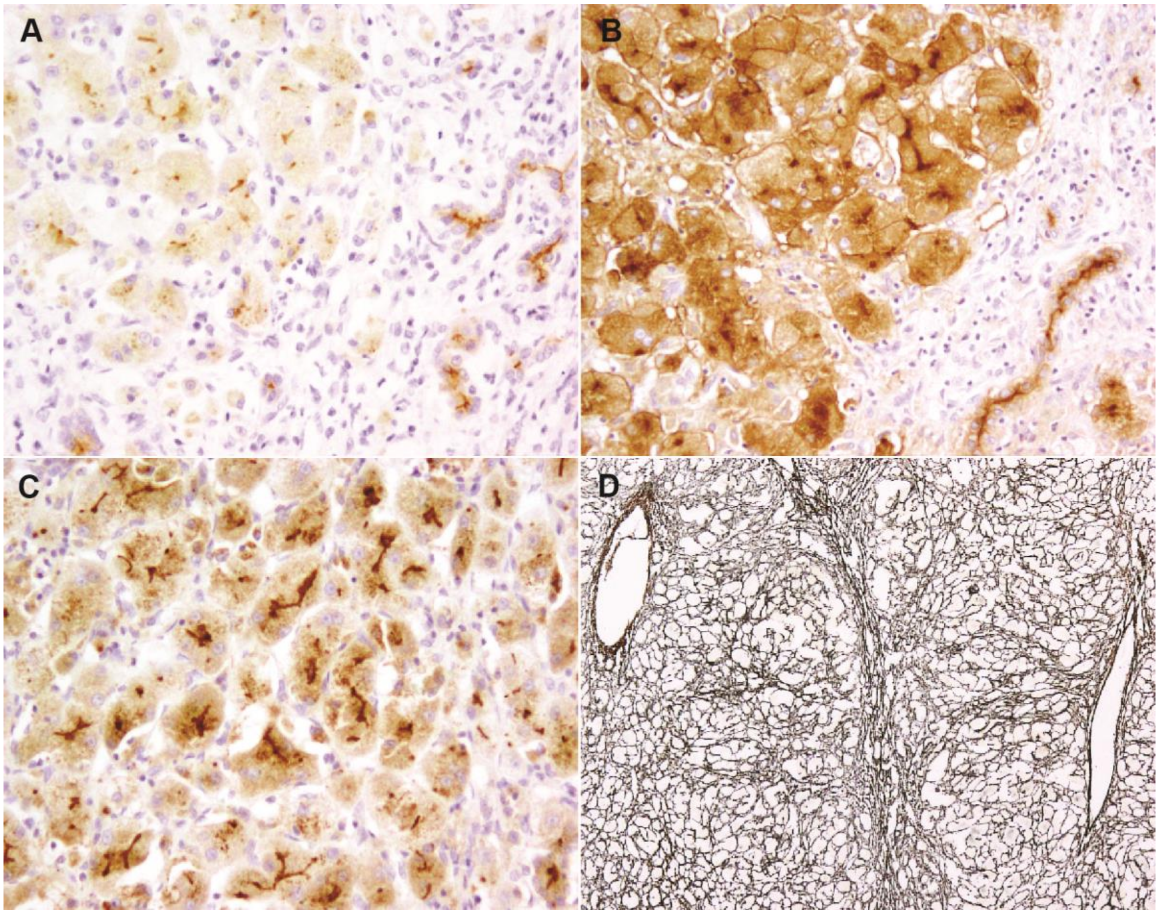

Supplementary Figure 2. Additional histological findings, explanted liver, Patient 1. Distorted bile-canaliculus network margins marked immunohistochemically for alanyl aminopeptidase, GGT, and MRP2 (A - C respectively; diaminobenzidine chromogen, haematoxylin counterstain; all original magnifications 200x). Perisinusoidal and perihepatocytic reticulin fibers are prominently increased (D; Gordon & Sweets ammoniacal silver; original magnification 40x).

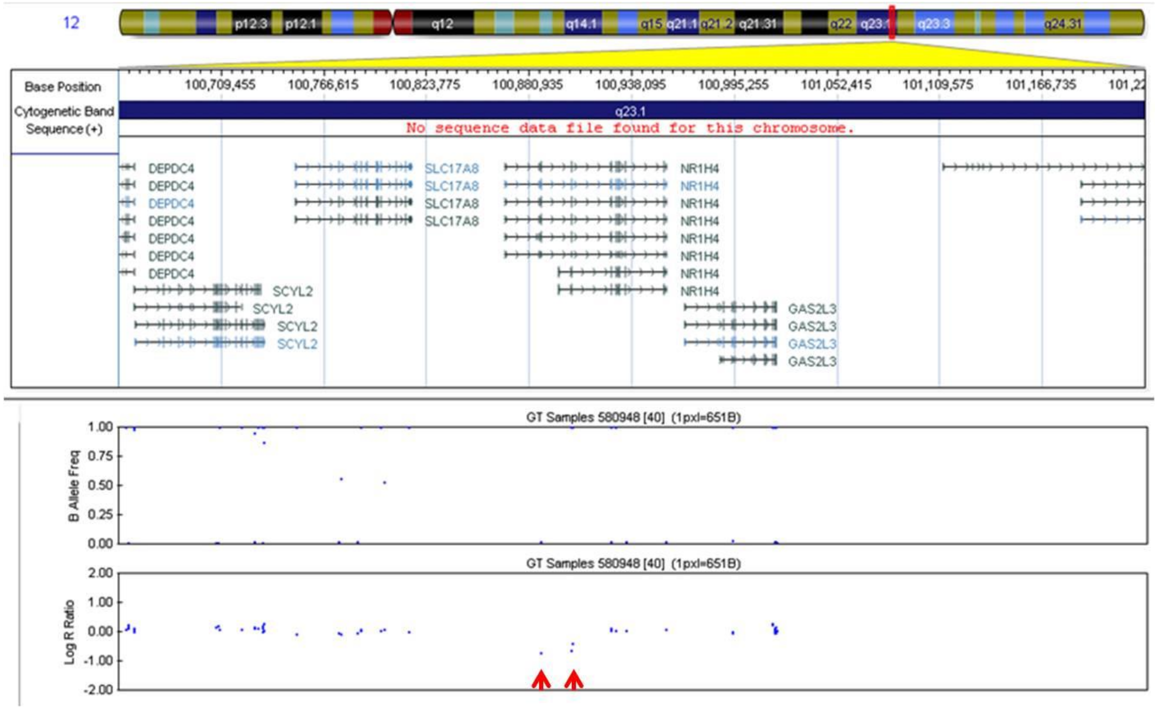

Supplementary Figure 3. cSNP analysis shows microdeletion at *NR1H4* in the proband (patient 4) of family 2. A snapshot of the cSNP analysis shows reduced signal intensities of three consecutive probes (red arrows), suggesting copy number loss at the *NR1H4* locus.

a

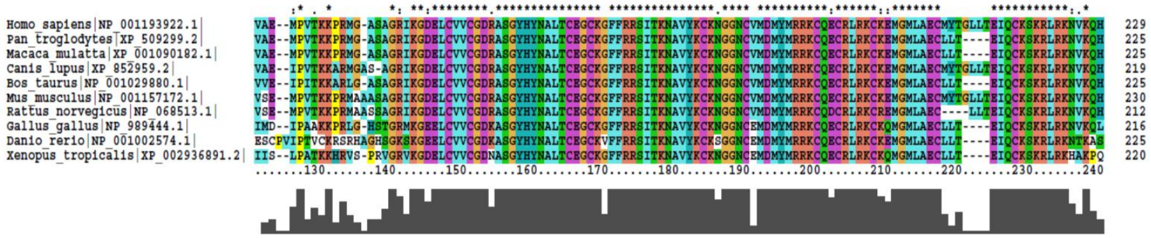

b

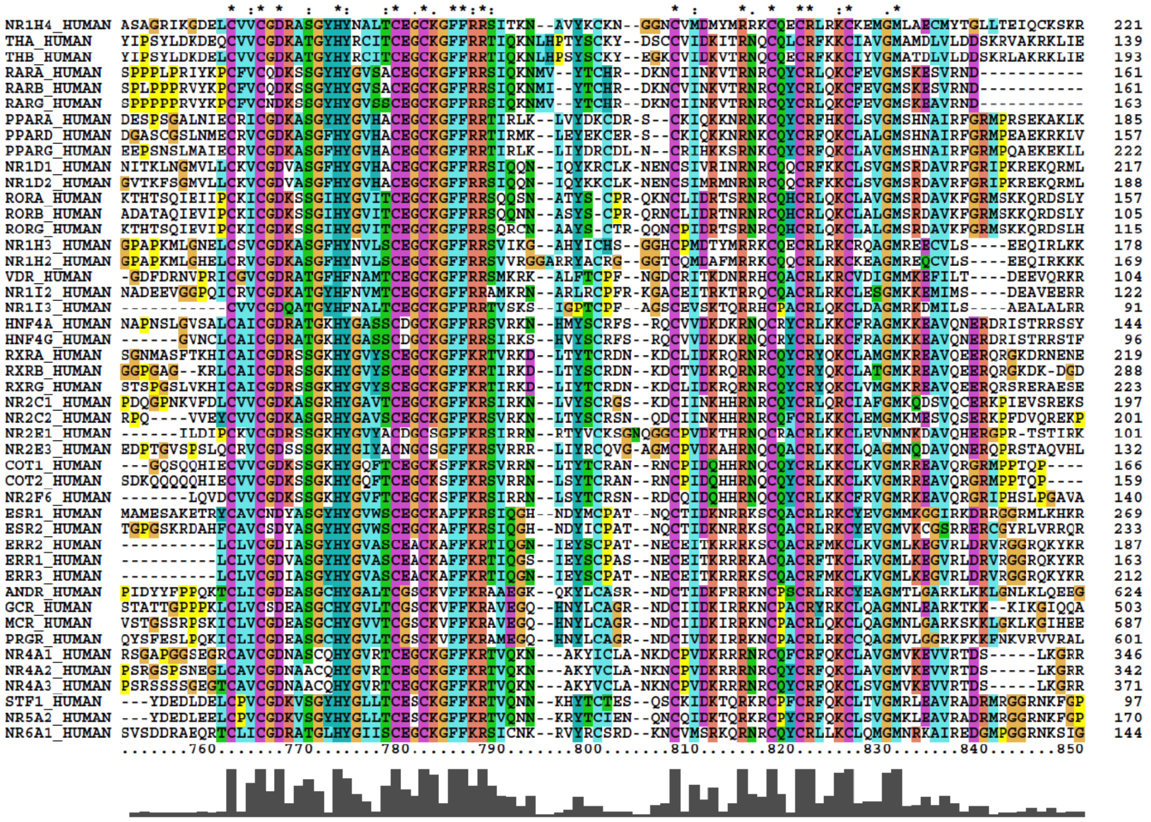

Supplementary Figure 4. DNA binding domain of FXR is highly conserved. a Amino acid sequence alignment of FXR DNA binding domains from different species. **b** Amino acid sequence alignment of all human nuclear receptors with DNA binding domains.

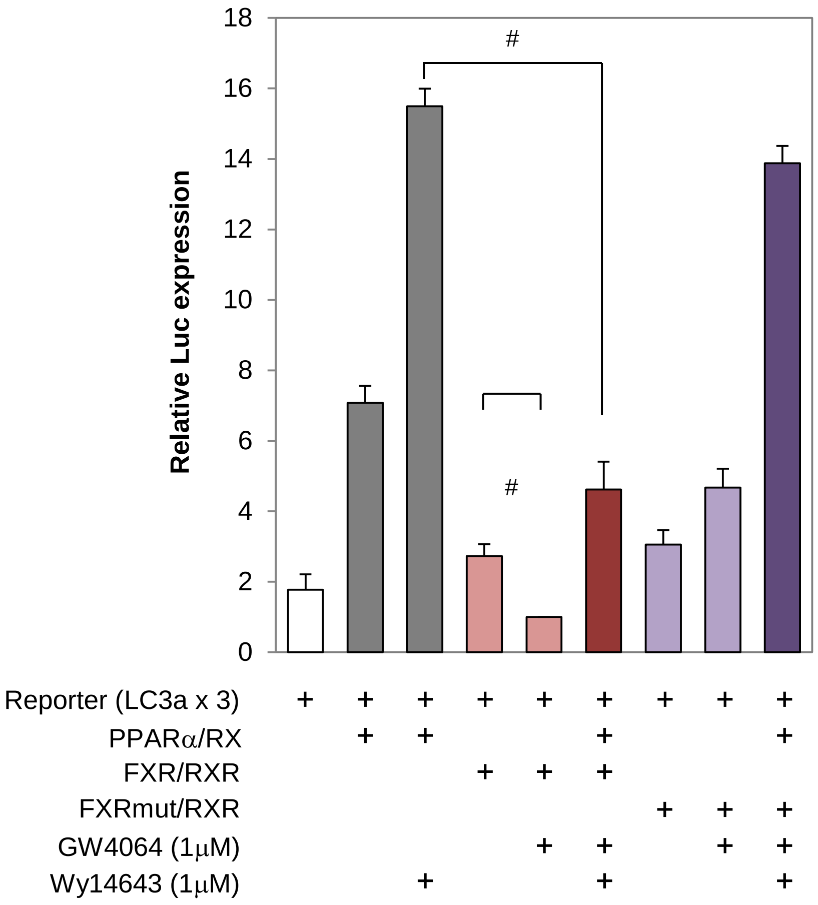

Supplementary Figure 5. Insertion mutant FXR is defective in DR-1 transrepression. AML12 cells were transfected with a reporter containing 3 copies of the DR-1 element from the LC3a gene, which was previously shown to be transactivated by PPARα and transrepressed by FXR[1], and the indicated expression vectors. The FXR agonist GW4064 decreased expression in the presence of FXR and RXR alone, and also in the presence of FXR/RXR and Wy14643 activated PPARα. Both responses were lost in the single amino acid Tyr139_Asn140insLys mutant FXR.

[1]Lee, J.M. *et al.* Nutrient-sensing nuclear receptors coordinate autophagy. *Nature* **516**, 112-5 (2014).

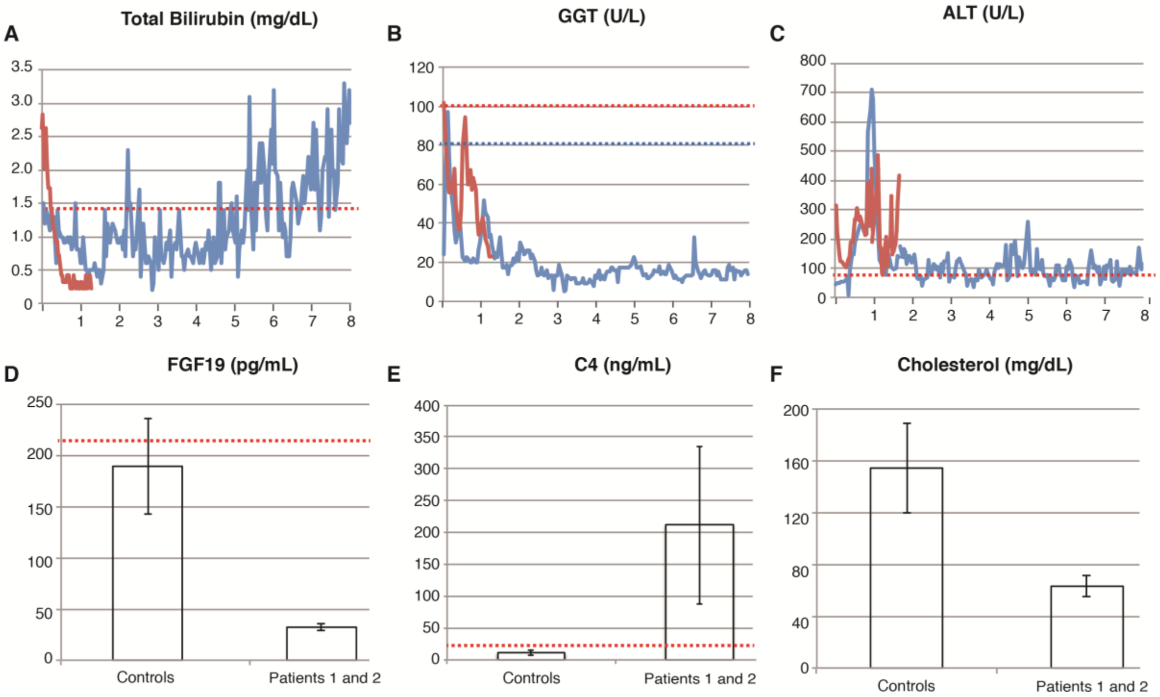

Supplementary Figure 6. Total bilirubin, GGT, ALT, C4, FGG19 and cholesterol values after liver transplant for patients in Family 1. A. total bilirubin values (mg/dL) for Patients 1 (blue) and 2 (red). X-axis, years after transplant. Dashed lines indicate upper limit of normal in the pediatric population. Notice developing conjugated hyperbilirubinemia in patient 1. **B.** GGT is stably within normal ranges for both patients. **C.** Patient 2 shows persistent moderate elevations in ALT and waxing and waning course; Patient 1 shows mild elevations. **D.** markedly decreased FGF19 levels (Patient 1, 11.1 pg/mL (assay sensitivity is 15.6 pg/mL); Patient 2, 29.4 pg/mL, **E.** increased C4 levels (Patient 1, 114 ng/mL; Patient 2, 322 ng/mL) and **F.** decreased cholesterol levels in both patients.

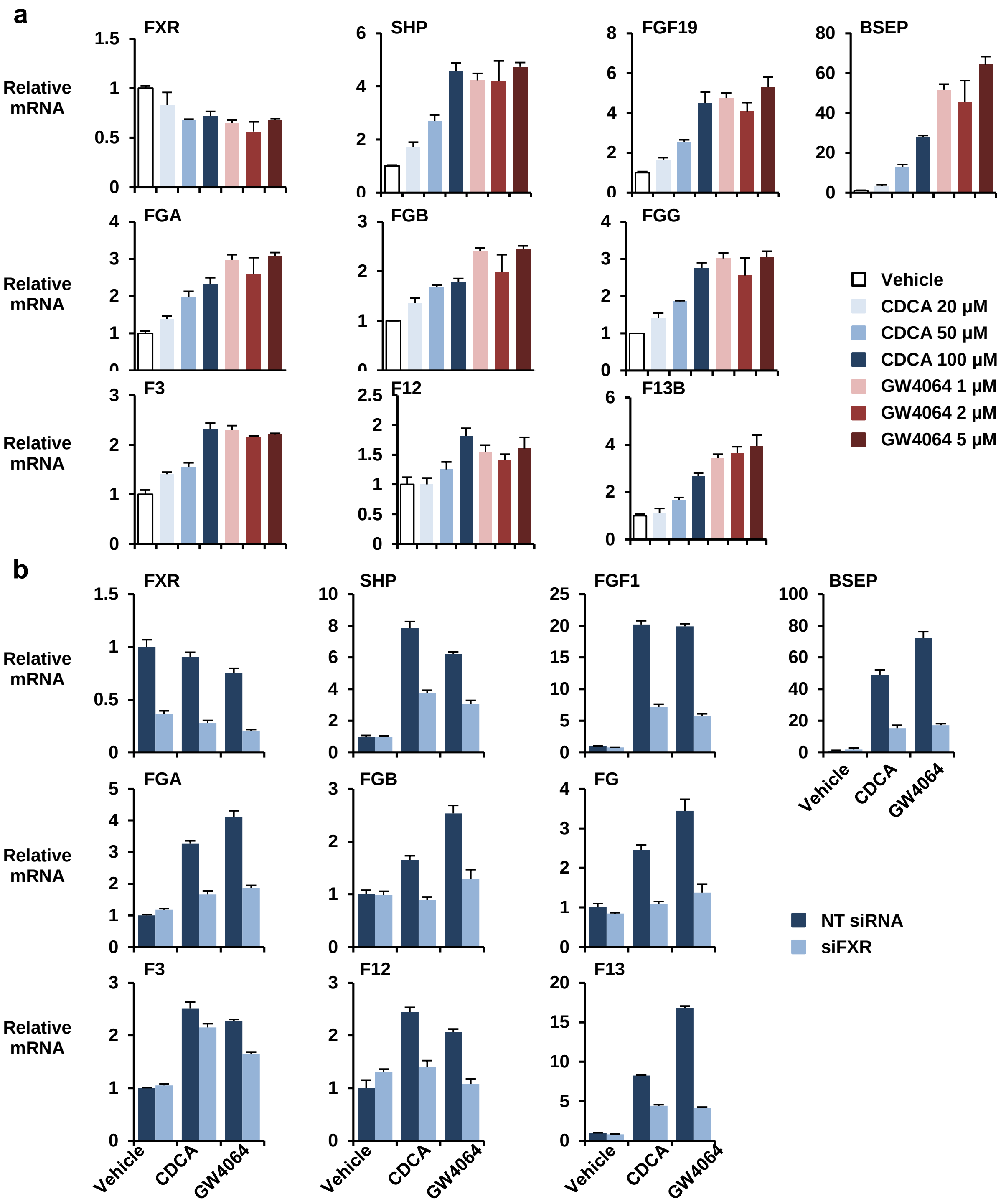

Supplementary Figure 7. FXR induces multiple components of the coagulation cascade. a. Huh7 cells were treated with FXR ligands (CDCA and GW4064) at

indicated concentrations and mRNA levels of coagulation core genes were assessed by real-time PCR. **b**. Huh7 cells were transfected with either non-targeting siRNA or FXR siRNA. After treatment of FXR ligands (CDCA, 100 μM; GW4064, 1 μM), gene expressions were analyzed by real-time PCR. All data were triplicate and represent mean ± s.e.m. BSEP=ABCB11; FGA=Fibrinogen alpha; FGB=Fibrinogen beta; FGG=Fibrinogen gamma; F3=Tissue Factor, Tissue Thromboplastin; F12=Coagulation Factor XII, Hageman Factor; F13B=Coagulation Factor XIII B Subunit, Fibrin Stabilizing Factor B Subunit. Primers in Supplementary Table 2.

Supplementary Table 1. Additional laboratory findings

	Family 1				Family 2		
	Patient 1		Patient 2		Patient 3		Patient 4
**Liver Biochemistry**	**Initial**	**Before OLT**	**Initial**	**Before OLT**	**Initial**	**Prior to death**	**Birth to death**
Total Bilirubin (nl <1.4 mg/dL)	23.8	29.4	16	21.1	14	35.6	3.9-14.1
Alkaline Phosphatase (nl <400 U/L)	627	515	586	1236	368		20-57
Albumin (nl 3.5-5.0 g/dL)	2.1	2.4	2.5	2.7	2.8	3.4	1.5-2.5**
Ammonia (umol/L)	71-95	95-165	64-93	56-125	47	233	37
Total Bile Acids (nl < 19.2)	93.7	NM	NM	NM	NM	NM	NM
Cholic acid (nl < 3.1)	47.7	NM	NM	NM	NM	NM	NM
Chenodeoxycholic acid (nl < 9.9)	44.5	NM	NM	NM	NM	NM	NM

NM; Not measured

** These values are not representative due to transfusions required for volume/losses and blood pressure support.

Supplementary Table 2
Primer Sets

Gene	Forward	Reverse
GAPDH	CCAGCAAGAGCACAAGAGGA	GAGATTCAGTGTGGTGGGGG
FXR	GCTTTGCTGAAAGGGTCTGC	CAGAATGCCCAGACGGAAGT
SHP	TCAAGTCCATTCCGACCAGC	AAGAAGGCCAGCGATGTCAA
FGF19	AGATCAAGGCAGTCGCTCTG	CGGATCTCCTCCTCGAAAGC
BSEP	GAGCCTGGTCATCTTGTGCT	TCTCCAGGGCCTGCTTATCT
FGA	CTGCCTGGTCCTAAGTGTGG	GCAGAAGGGCCAGTCTGAAT
FGB	ACCATGGAAAAGCCACCACT	CGTTTCTATGGGCAACAGGC
FGG	CACACAGTCTGCCATCCCATA	ACTTGTCAGCTTCAGGTCCC
F3	ACTCCCCAGAGTTCACACCT	TCCCGGAGGCTTAGGAAAGT
F12	AGCTGAAGAGCACACAGTCG	CGGCCCTTGTGGGTACATTT
F13b	GAATGCAATGTGACAGAGGGC	TGACTCCTCTTTCTGCCATTCA
